# Current findings for recurring mutations in acute myeloid leukemia

**DOI:** 10.1186/1756-8722-4-36

**Published:** 2011-09-14

**Authors:** Shinichiro Takahashi

**Affiliations:** 1 Division of Molecular Hematology, Kitasato University Graduate School of Medical Sciences and Division of Hematology, Kitasato University School of Allied Health Sciences, 1-15-1 Kitasato, Minami-ku, Sagamihara 252-0373, Japan

**Keywords:** gene mutations, acute myeloid leukemia, cooperative interactions

## Abstract

The development of acute myeloid leukemia (AML) is a multistep process that requires at least two genetic abnormalities for the development of the disease. The identification of genetic mutations in AML has greatly advanced our understanding of leukemogenesis. Recently, the use of novel technologies, such as massively parallel DNA sequencing or high-resolution single-nucleotide polymorphism arrays, has allowed the identification of several novel recurrent gene mutations in AML. The aim of this review is to summarize the current findings for the identification of these gene mutations (Dnmt, TET2, IDH1/2, NPM1, ASXL1, etc.), most of which are frequently found in cytogenetically normal AML. The cooperative interactions of these molecular aberrations and their interactions with class I/II mutations are presented. The prognostic and predictive significances of these aberrations are also reviewed.

## Introduction

The identification of mutations in certain genes, such as the fms-related tyrosine kinase 3 (FLT3), CCAAT/enhancer binding protein alfa (C/EBPα), runt-related transcription factor 1 (RUNX1), myeloid-lymphoid or mixed lineage leukemia (MLL), Wilms tumor (WT1) and nucleophosmin (NPM) 1 genes, in acute myeloid leukemia (AML) has significantly improved our understanding of leukemogenesis [1-4]. This is particularly the case for patients with normal cytogenetics, who comprise the largest subgroup (approximately 45%) of AML patients [5-7]. In fact, assessments of the presence of internal tandem duplications in the FLT3 receptor gene (FLT3-ITD) [8] and mutations in the NPM1 gene [4] are currently routine practices in guiding therapeutic decisions in AML patients with a normal karyotype [9]. Recent studies have revealed prevalent mutations, such as DNA methyltransferase (Dnmt) 3a mutations [10], ten-eleven-translocation oncogene family member 2 (TET2) [11] mutations and isocitrate dehydrogenase (IDH) 1 gene mutations [3], using novel technologies like high-throughput massively parallel DNA sequencing [12]. Indeed, AML is increasingly subclassified as a unique entity in the 2008 revision of the World Health Organization classification of myeloid neoplasms and acute leukemia [13], based on specific recurring genetic abnormalities that can predict the prognosis and response to therapy [7].

**Figure 1:**
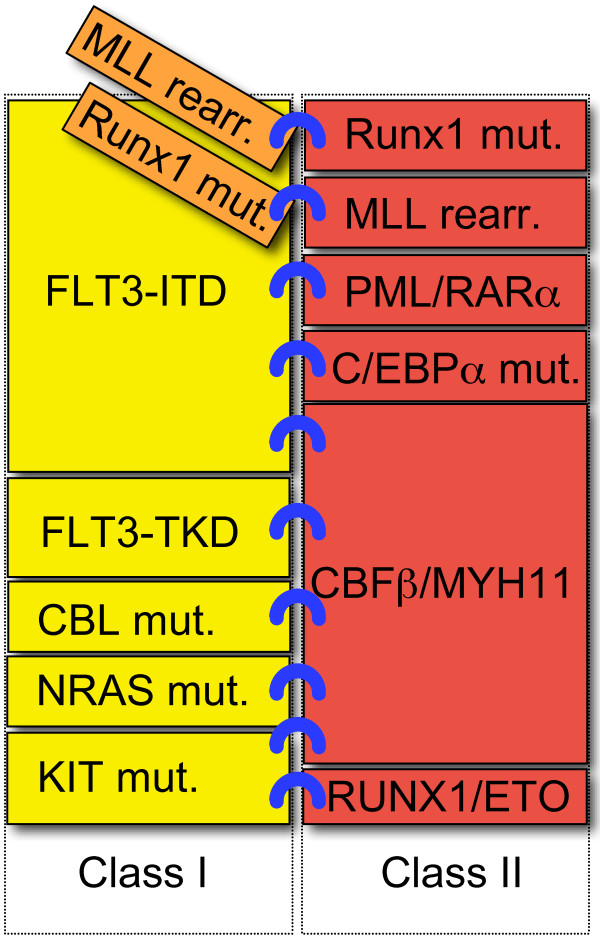
**The model of the "classical" class I and class II mutations in AML**. This model comprises class I mutations that activate signal transduction pathways and confer a proliferation advantage on hematopoietic cells, and class II mutations that affect transcription factors and primarily serve to impair hematopoietic differentiation [15,16]. The development of AML is a multistep process that requires at least these two genetic abnormalities for the development of the disease. Class I mutations are shown in yellow boxes and class II mutations are in red boxes. The combination of each mutation is demonstrated as blue rings. Runx 1 mutations and MLL rearrangements may be exception in this model, as shown in orange boxes, since co-occurrence is observed between these two mutations.

AML development is considered to be a multistep process that requires the collaboration of at least two classes of mutations to obtain full-blown leukemia. Almost a decade ago, Gilliland and Griffin [14] presented a paradigm model for this process, designated the "two-hit model". This model comprises class I mutations that activate signal transduction pathways and confer a proliferation advantage on hematopoietic cells, and class II mutations that affect transcription factors and primarily serve to impair hematopoietic differentiation [15,16]. Mutations leading to activation of the receptor tyrosine kinase (RTK) FLT3, c-kit (KIT) and Ras signaling pathway are considered to be class I mutations. Recurring chromosomal aberrations such as t(8; 21), inv(16) and t(15; 17), which generate fusion transcripts of RUNX1/ETO, CBFβ/MYH11 and PML/RARα, respectively, fall into class II mutations. Not only chromosomal abnormalities but also mutations of the transcription factors RUNX1, C/EBPα and MLL are classified into this group. These "classical" class I and class II mutations are presented in Figure 1. However, most of the newly identified genetic alterations, such as those in Dnmt, TET2, IDH1, IDH2, NPM1, ASXL1, which are addressed mainly in this review, have not been classified because the consequences of these mutations have not been identified. In this review, each of these "unclassified mutations" is detailed individually. The author summarizes the current findings of these novel gene mutations, most of which are frequently found in cytogenetically normal AML (CN-AML). The cooperative interactions of the molecular aberrations are also presented.

## Dnmt mutations

Dnmts are enzymes that catalyze the addition of a methyl group to the cytosine residue of CpG dinucleotides. Aberrant CpG island methylation has long been hypothesized to contribute to the pathogenesis of cancer [17]. Recently, using massively parallel DNA sequencing [12], Ley *et al. *[10] identified a somatic mutation in Dnmt3a by sequencing 116.4 billion base pairs of the sequence with 99.6% diploid coverage of the genome of cells from an AML patient with a normal karyotype. Further analyses revealed the presence of Dnmt3a mutations in 62 of 281 AML patients (22.1%). These mutations were highly enriched in a group of patients with an intermediate-risk cytogenetic profile, as well as mutations in FLT3 (either ITD or tyrosine kinase domain [TKD] mutations), NPM1 and IDH1. The median overall survival (OS) among patients with Dnmt3a mutations was significantly shorter than that among patients without such mutations (12.3 vs. 41.1 months, p < 0.001) [10] (Table 1). Walter *et al. *[18] also described relatively frequent mutations in myelodysplastic syndrome (MDS). They carried out sequencing for 150 patients with MDS, and identified 13 heterozygous mutations with predicted translational consequences in 12 patients (8.0%). Yan *et al. *[19] discovered Dnmt3a mutations in 23 of 112 cases (20.5%) with the M5 subtype of AML. They revealed that, although Dnmt3a mutations do not dramatically alter global DNA methylation levels in AML genomes [10], there were alterations of specific gene DNA methylation patterns and/or gene expression profiles, such as HOXB genes, in samples with Dnmt3a mutations compared with those without such changes [19]. Consistent with these observations, the Dnmt3a mutations, which frequently occurred in arginine (R) 882, caused reduced enzymatic activity *in vitro *[19].

**Table 1:** Clinical features of gene mutations in AML (unclassified mutations)

Gene	Clinical Features	**Selected Ref**.	Frequency
DNMT3a	The median OS among patients with Dnmt3a mutations was significantly shorter than wild type patients.	[10]	22.1% in AML
TET2	In the European Leukemia Net (ELN) favorable-risk group, TET2-mutated patients had shorter EFS (EFS; p < 0.001) because of a lower CR rate (p = 0.007), and shorter DFS (p = 0.003), and also had shorter OS (p = 0.001) compared with TET2-wild type patients. (TET2 mutations were not associated with outcomes in the ELN intermediate-I-risk group.)	[21]	23% in CN-AML
IDH1	IDH 1 mutation was associated with normal cytogenetics, a higher RR and a shorter OS. Prognosis was adversely affected by IDH1 mutations with trend for shorter OS (p = 0.110), a shorter EFS (p < 0.003) and a higher cumulative risk for relapse (p = 0.001). Clear prevalence in intermediate risk karyotype group (10.4%, p < 0.001).	[25-27]	6.6-9.6% in AML
IDH2	In IDH2 mutation CN-AML patients, there is a higher risk of induction failure, a higher RR and shorter OS.	[25,26]	3.0-8.7% in AML
NPM1	The analysis of the clinical impact in 4 groups (NPM1 and FLT3-ITD single mutants, double mutants, and wild-type for both) revealed that patients having only an NPM1 mutation had a significantly better OS and DFS and a lower cumulative incidence of relapse.	[28,29]	27.5-35.2% in AML 45.7-53% in CN-AML
ASXL1	Patients with ASXL1 mutations had a shorter OS than patients without, but the mutation was not an independent adverse prognostic factor in multivariate analysis.	[39]	10.8% in AML
WT1	Multivariate analysis demonstrated that the WT1 mutation was an independent poor prognostic factor for OS and RFS among total patients and the CN-AML group.	[41,42]	8.3-10.7% in CN-AML 6.8% in de novo non-M3 AML

## TET2 mutations

In 2009, Delhommeau *et al. *[11] conducted high-resolution single-nucleotide polymorphism and comparative genomic hybridization arrays to identify a candidate tumor-suppressor gene common to patients with MDS, myeloproliferative disorders and AML. They identified inactivating mutations of the TET2 gene in about 15% of patients with various myeloid malignancies, such as MDS (19%), myeloproliferative disorders (12%), secondary AML (24%) and chronic myelomonocytic leukemia (CMML) (22%) [11]. TET2 can convert 5-methylcytosine (5-mC) to 5-hydroxymethylcytosine (5-hmC) [20], which to be an intermediate in DNA demethylation. Bone marrow samples from patients with TET2 mutations displayed uniformly low levels of 5-hmC in genomic DNA compared with bone marrow samples from healthy controls [20]. Metzeler *et al. *[21] recently analyzed 427 patients with CN-AML, and revealed that TET2 mutations were detected in 95 of 418 (23%) of the patients, and associated with older age (p < 0.001) and higher pretreatment white blood cell counts (p = 0.04) compared with wild-type TET2. IDH1 and IDH2 mutations were less frequent in TET2-mutated patients than in TET2-wild-type patients (p < 0.001), suggesting that these mutations are mutually exclusive. They also observed a trend toward a higher prevalence of C/EBPα mutations among TET2-mutated patients (p = 0.07) [21]. In the European Leukemia Net (ELN) favorable-risk group (patients with CN-AML who have mutated CEBPα and/or mutated NPM1 without FLT3-ITD), TET2-mutated patients had shorter event-free survival (EFS) (p < 0.001), because of a lower complete remission (CR) rate (p = 0.007), shorter disease-free survival (DFS) (p = 0.003) and shorter OS (p = 0.001) compared with TET2-wild-type patients (Table 1). TET2 mutations were not associated with outcomes in the ELN intermediate-I-risk group (CN-AML with wild-type CEBPα and wild-type NPM1 and/or FLT3-ITD). In multivariate models, TET2 mutations were associated with shorter EFS (p = 0.004), lower CR rate (p = 0.03) and shorter DFS (p = 0.05) only among favorable-risk CN-AML patients [21]. Abdel-Wahab *et al. *[22] evaluated the mutational statuses of TET1, TET2 and TET3 in myeloproliferative neoplasms (MPNs), CMML and AML. They identified TET2 mutations in 27 of 354 MPN patients (7.6%), 29 of 69 CMML patients (42%), 11 of 91 AML patients (12%) and 1 of 28 M7 AML patients (3.6%). Although they identified several single nucleotide polymorphisms in TET1 and TET3, they did not identify somatic TET1 or TET3 mutations in 96 MPN patients examined.

## IDH1 and IDH2 mutations

Mardis *et al. *[3] used massively parallel DNA sequencing to obtain a very high level of coverage of a primary, cytogenetically normal, *de novo *genome for AML with minimal maturation (AML M1) and a matched normal skin genome, and identified 12 acquired mutations within the coding sequences of genes and 52 somatic point mutations in conserved or regulatory portions of the genome. Many of these were mutations that had already been identified, such as those in NRAS and NPM1, but they also found novel mutations of the *IDH1 *gene [3]. They further found that *IDH1 *gene mutations were present in 15 of 187 AML genomes and strongly associated with a normal cytogenetic status. IDH1 and IDH2 function at a crossroads involving cellular metabolism in lipid synthesis, cellular defense against oxidative stress, oxidative respiration and oxygen-sensing signal transduction [23]. Recently, AML patients harboring IDH1 and IDH2 mutations were found to show aberrant hypermethylation [24]. In fact, these mutations led to the production of an abnormal cellular metabolite, 2-hydroxyglutarate, which can inhibit the hydroxylation of 5-mC by TET2 [24]. Consistent with Metzeler *et al. *[21], IDH1 and IDH2 mutations were mutually exclusive with TET2 mutations (p = 0.009) [24]. Boissel *et al. *[25] analyzed the prognosis of patients with IDH1 mutations and IDH2 mutations in a cohort of 520 adults with AML homogeneously treated in the French Acute Leukemia French Association (ALFA)-9801 and -9802 trials. The prevalences of IDH1 mutations and IDH2 mutations were 9.6% and 3.0%, respectively, and the mutations were mostly associated with CN-AML. In patients with CN-AML, IDH1 mutations were associated with higher risk of relapse (RR) and shorter OS (Table 1). In CN-AML patients with IDH2 mutations, they observed a higher risk of induction failure, higher RR and shorter OS. Similar results were reported by Paschka *et al. *[26], who evaluated 805 adults with AML enrolled in the German-Austrian AML study group, and found IDH mutations in 129 patients (16.0%), IDH1 mutations in 61 patients (7.6%) and IDH2 mutations in 70 patients (8.7%). These two reports both suggest the presence of interactions between IDH mutations and the genotype of mutated NPM1 without FLT3-ITD. They also both demonstrated that IDH mutations in AML are associated with a poor prognosis. Schnittger *et al. *[27] conducted a larger study. They analyzed IDH1R132 mutations in 1414 AML patients, and detected IDH1 mutations in 6.6% of the patients, with a clear prevalence in the intermediate-risk karyotype group (10.4%; p < 0.001). They also showed that IDH1 mutations had strong associations not only with NPM1 mutations (p < 0.001), but with MLL-partial tandem duplicaton (PTD) as well (p = 0.020) (Table 1). In addition, they revealed that the prognosis was adversely affected by IDH1 mutations with trends for shorter OS (p = 0.110), shorter EFS (p < 0.003) and higher cumulative RR (p = 0.001) (Table 1).

## NPM1 mutations

Falini *et al. *[4] described abnormal localization of NPM1 in AML patients. Cytoplasmic NPM1 was detected in 208 of 591 specimens (35.2%) from patients with primary AML, but not in 135 secondary AML specimens or in 980 hematopoietic or extrahematopoietic neoplasms other than AML [4]. Thiede *et al. *[28] performed a larger study. They investigated 1485 AML patients for NPM1 exon 12 mutations, and found that the C-terminus of this protein was mutated in approximately 27.5% of the patients. NPM1 mutations were more prevalent in patients with a normal karyotype (324 of 709; 45.7%) than in patients with karyotype abnormalities (58 of 686; 8.5%; p < 0.001). They suggested that NPM1 mutations are strongly associated with FLT3-ITD mutations in patients with a normal karyotype (mutated NPM1/FLT3-ITD, 43.8% vs. wild-type NPM1/FLT3-ITD, 19.9%; p < 0.001) [28]. Analyses of the clinical impacts in four groups (NPM1 single mutants, FLT3-ITD single mutants, NPM1/FLT3-ITD double mutants, and wild-type for both) revealed that patients with only NPM1 mutations had significantly better OS and DFS and a lower cumulative incidence of relapse [28] (Table 1). Schlenk *et al. *[29] focused their analyses on CN-AML patients. Among 872 patients examined, they found that 53% of the patients had NPM1 mutations. In addition, 31% had FLT3-ITD, 11% had FLT3-TKD, 13% had C/EBPα mutations, 7% had MLL-PTD and 13% had NRAS mutations. They further demonstrated that FLT3-ITD (p < 0.001) and FLT3-TKD mutations (p = 0.03) were significantly associated with NPM1 mutations, while NRAS mutations were not (p = 0.46).

NPM1 is thought to have relevant roles in diverse cellular functions, including ribosome biogenesis, centrosome duplication, DNA repair and response to stress [30]. NPM1 is also involved in the functions of p53 and p19ARF [31,32]. Li *et al. *[33] demonstrated that wild-type NPM1 protects hematopoietic cells against p53-induced apoptosis under conditions of cellular stress. Therefore, it is possible that failure of the mutated NPM1 to protect cells may make them more sensitive to high-level genotoxic stress induced by chemotherapy. Consequently, it is possible to speculate that patients with mutated NPM1 have a better prognosis.

## ASXL1 mutations

The additional sex comb-like 1 (ASXL1) gene belongs to a family with three identified members that encode poorly characterized proteins involved in the regulation of chromatin remodeling. The ASXL proteins contain a C-terminal plant homeodomain (PHD) finger and belong to the polycomb and trithorax complexes that regulate the genetic program of stem cells. ASXL1 is involved in the regulation of histone methylation by cooperation with heterochromatin protein-1 to modulate the activity of lysine-specific demethylase (LSD) 1 [34], a histone demethylase for H3K4 and H3K9 that is also important for global DNA methylation [35]. However, the hematopoietic function of ASXL1 is still unclear, since an ASXL1 knockout mouse model shows only a mild hematopoietic phenotype [36]. In 2009, Gelsi-Boyer *et al. *[37] first identified mutations of ASXL1 in 40 MDS/AML samples using high-density comparative genomic hybridization arrays. They found mutations in the *ASXL1 *gene in 4 of 35 MDS patients (11%) and 17 of 39 CMML patients (43%). Another study identified mutations of the *ASXL1 *gene in 12 of 63 (19%) secondary AML patients transformed from MPN [38]. Recently, Chou *et al. *[39] examined *ASXL1 *gene mutations in exon 12 in 501 adults with *de novo *AML. ASXL1 mutations were detected in 54 patients (10.8%), with 8.9% among patients with a normal karyotype and 12.9% among patients with abnormal cytogenetics. The mutations were closely associated with older age, male sex, isolated trisomy 8, RUNX1 mutations, and expression of human leukocyte antigen-DR and CD34, but inversely associated with t(15; 17), complex cytogenetics, FLT3-ITD, NPM1 mutations, WT1 mutations and expression of CD33 and CD15. Patients with ASXL1 mutations had shorter OS than patients without such mutations, but the mutations were not an independent adverse prognostic factor in a multivariate analysis [39] (Table 1).

## WT1 mutations

Although mutations of WT1 were first discovered in hematological malignancies more than a decade ago, the precise roles of WT1 in normal and malignant hematopoiesis remain elusive [40]. It has been implicated in the regulation of cell survival, proliferation and differentiation, and may function as both a tumor suppressor and an oncogene [40]. Paschka *et al. *[41] analyzed 196 adults aged < 60 years with newly diagnosed primary CN-AML, who were treated similarly with Cancer and Leukemia Group B (CALGB) protocols 9621 and 19808, for WT1 mutations in exons 7 and 9. As a result, 21 patients (10.7%) harbored WT1 mutations. The patients with WT1 mutations had worse DFS (p < 0.001) and OS (p < 0.001) than patients with wild-type WT1. Subsequently, Hou *et al. *[42] examined the clinical implications of WT1 mutations in 470 *de novo *non-M3 AML patients aged ≥ 15 years, and their stability during the clinical course. WT1 mutations were identified in 6.8% of the total patients and 8.3% of the younger patients with CN-AML. The WT1 mutations were closely associated with younger age (p < 0.01), French-American-British M6 subtype (p = 0.006) and t(7; 11)(p15; p15) (p = 0.003). A multivariate analysis demonstrated that WT1 mutations comprised an independent poor prognostic factor for OS and relapse-free survival (RFS) among the total patients and the CN-AML patients. In addition, among 32 patients with WT1 mutations, the most frequently associated molecular event was FLT3-ITD (9 cases) [42]. Becker *et al. *[43] investigated 243 older (≥ 60 years) primary CN-AML patients, and found that WT1-mutated patients (7%) had more frequent FLT3-ITD (p < 0.001) and shorter OS (p = 0.08) compared with WT1-wild-type patients. The clinical features of the unclassified mutations are summarized in Table 1.

## Class I mutations (FLT3, PTPN11, NRAS, KIT and CBL mutations)

### FLT3 mutations

FLT3 is a type III RTK and since the first description [44], numerous studies have confirmed and extended the findings that FLT3 mutations are currently one of the most frequent single mutations identified in AML. ITD mutations of the *FLT3 *gene occur in approximately 21-24% of adult AML patients [14,28] while activating point mutations of the FLT3-TKD, mainly at Asp 835, are found in approximately 5-7% of AML patients [1,45-48]. The most significant impacts of an ITD are its associations with a higher leukocyte count, increased RR, decreased DFS and decreased OS, which have been reported in most studies of children and adults aged < 60 years [49]. Several groups found that an ITD is a significant predictive factor for an adverse outcome in multivariate analyses [49-52] (Table 2). Bacher *et al. *[46] performed a large study involving 3082 patients with newly diagnosed AML, and analyzed the mutational status and clinical significance of the FLT3-TKD. They observed FLT3-TKD mutations in 147 patients (4.8%). Unlike FLT3-ITD, the prognosis was not influenced by FLT3-TKD mutations in a total cohort of 1720 cases where follow up-data were available (97 mutated FLT3-TKD cases and 1623 wild-type FLT3 cases) (Table 2). In addition, Ozeki *et al. *[53] reported that even in patients with wild-type FLT3, a clear tendency for worse OS was found in patients with high FLT3 expression (5 of 86 patients without FLT3-ITD). Another group observed a similar result for a tendency toward lower OS (12 of 24 patients, p = 0.059) and EFS (7 of 20 patients, p = 0.087) in the group with high FLT3 expression [54] (Table 2).

**Table 2:** Clinical features of gene mutations in AML (class I mutations)

Gene	Clinical Features	**Selected Ref**.	Frequency
FLT3 -ITD	Association with a higher leukocyte count, increased RR, decreased DFS, and decreased OS.	[14,28,49-52]	21-24% in AML
-TKD	Prognosis was not influenced.	[46]	5-7% in AML
-WT	Clear tendencies for worse OS and EFS were found in patients with high FLT3 expression.	[53,54]	
PTPN11	No prognostic significance. However, subgroup analysis did reveal that the PTPN11 mutation was a poor risk factor for OS of AML patients who did not have NPM1 mutations.	[62]	5.1% in AML
NRAS	No significant prognostic impact for OS, EFS and DFS.	[64]	10.3% in AML
KIT	Adversely affect OS in AML with inv(16). Adverse impact of mutation of KIT on RR in t(8; 21)AML. KIT mutations had an independent negative impact on OS and EFS in patients with t(8;21) but not in patients with a normal karyotype.	[66,68,69]	1.7% in AML 22-45% in t(8; 21) 29-48% in inv(16)
CBL	n. d.	[72,73]	1.1% in AML/MDS 16% in inv(16)AML

FLT3-ITD mutations are correlated with certain cytogenetic subgroups. Among acute promyelocytic leukemia patients with PML-RARα, it was reported that 30-50% of patients had FLT3 mutations [55-57]. In addition, frequent (88~90%) co-occurrence was reported in patients with t(6; 9) and FLT3-ITD [55,58]. Similarly, FLT3-ITD was frequently found in patients with MLL-PTD [59]. The rate of MLL-PTD in FLT3-ITD-positive patients was significantly higher than that in FLT3-ITD-negative patients [16 of 184 (8.7%) vs. 32 of 772 (4.1%); p = 0.025] [59]. In analyses involving 353 adult *de novo *AML patients, Carnicer *et al. *[60] found cooperative mutations of FLT3-TKD with CBFβ/MYH11 rearrangement (4 of 15 patients) and C/EBPα with FLT3-ITD (2 of 82 patients). Collectively, FLT3 mutations play a key role in leukemogenesis by functionally cooperating with other molecules.

### PTPN11 mutations

SHP-2 is a cytoplasmic protein tyrosine phosphatase (PTP) that contains two Src homology 2 (SH2) domains. Although PTPs are generally considered to be negative regulators, SHP-2 is unusual in that it promotes the activation of the RAS-MAPK signaling pathway through receptors for various growth factors and cytokines. Mutations in the protein tyrosine phosphatase non-receptor type 11 (PTPN11), as the human *SHP-2 *gene, have been shown to produce dominant active mutants *in vitro *[61]. Hou *et al. *[62] investigated the prevalence and clinical relevance of mutations of PTPN11 and their associations with other genetic changes in 272 consecutive patients with primary AML. Among 14 patients with PTPN11 mutations (5.1%), none had FLT3-ITD. On the other hand, 6 of 13 patients with PTPN11 mutations had concurrent NPM1 mutations (46.2%) [62], suggesting that PTPN11 is a class I mutation molecule similar to the case for FLT3. They further revealed that PTPN11 mutations had no prognostic significance. The CR rate (75% vs. 62%) and median OS (13 ± 8.95 vs. 25.5 ± 6.54 months) were similar between patients with and without PTPN11 mutations. However, subgroup analyses did reveal that PTPN11 mutations comprised a poor risk factor for OS of AML patients without NPM1 mutations (p = 0.001) [62] (Table 2).

### NRAS mutations

Ras oncogenes encode a family of guanine nucleotide-binding proteins that regulate signal transduction upon binding to a variety of membrane receptors, including KIT and FLT3, and play important roles in proliferation, differentiation and apoptosis [63]. There are three functional *RAS *genes: *NRAS*, *KRAS *and *HRAS*. The *RAS *genes, especially *NRAS*, are frequently affected by mutations in AML. Bacher *et al. *[64] analyzed 2502 patients with AML, and found that 257 patients (10.3%) had NRAS mutations. The subgroups with inv(16) and inv(3) showed significantly higher frequencies of NRAS mutations. In contrast, NRAS mutations were significantly underrepresented in t(15; 17) (2 of 102; 2%; p = 0.005). They did not find significant prognostic impacts of NRAS mutations for OS, EFS and DFS (Table 2).

### KIT mutations

KIT is a member of the type III RTK family, and ligand-independent activation of KIT can be caused by gain-of-function mutations that have been reported in core binding factor (CBF) leukemia, and AML subgroups with inv(16) and t(8; 21) [65-67], which result in expression of the abnormal fusion genes CBFβ/MYH11 and RUNX1/ETO, respectively. Paschka *et al. *[68] analyzed 61 adults with CBF leukemia for KIT mutations. Among patients with inv(16), 29.5% had KIT mutations. Among patients with t(8; 21), 22% had KIT mutations. Mutations of the *c-kit *gene in both exon 17 and exon 8 appeared to adversely affect OS in AML with inv(16). They also observed an adverse impact of KIT mutations on RR in t(8; 21) AML patients (Table 2). Cairoli *et al. *[65] reported that among 42 patients with t(8;21), 19 patients (45.2%) had KIT mutations, whereas among 25 patients with inv(16), 12 patients (48.0%) had KIT mutations. Schnittger *et al. *[66] analyzed 1940 randomly selected AML patients and revealed that 33 patients (1.7%) were positive for KIT mutations in codon D816. Of these 33 patients, 8 patients (24.2%) had t(8; 21), which was significantly higher compared with the subgroup without D816 mutations. They revealed that KIT mutations had independent negative impacts on the median OS (304 vs. 1836 days; p = 0.006) and median EFS (244 vs. 744 days; p = 0.003) in patients with t(8; 21) but not in patients with a normal karyotype (Table 2). They also revealed that other activating mutations, like FLT3 and NRAS mutations, were very rarely detected in t(8; 21) leukemia patients. On the contrary, in an analysis of 99 patients with t(8; 21), Kuchenbauer *et al. *[69] reported that the frequent molecular aberrations with t(8; 21) were not only KIT D816 mutations (3 of 23 patients; 13%) but also NRAS mutations (8 of 89 patients; 8.9%). Although the co-occurrence of NRAS mutations and AML1/ETO expression remains elusive, all of these reports suggest that KIT mutants play important roles in CBF leukemia, with negative impacts on the clinical course [65,66,68,69].

### CBL mutations

The *Casitas B-cell lymphoma *(*CBL*) gene gives rise to the CBL protein, which has ubiquitin ligase activity and targets a variety of tyrosine kinases for degradation by ubiquitination [70]. CBL proteins also associate with the endocytic machinery and are thus important for the termination of RTK signaling [70]. CBL mutations were able to inhibit FLT3 internalization and ubiquitination [71]. *In vitro *experiments confirmed constitutive activation of the FLT3 pathway by the CBL mutants, and the phenotype of the altered cells resembled the phenotype of FLT3-mutated cells [72]. Reindl *et al. *[72] identified *c-CBL *gene exon 8/9 deletion mutants in 1.1% of 279 patients with AML/MDS. All the patients with CBL mutations had CBF leukemia and chromosome 11q abnormalities [72]. In a series of 37 patients with newly diagnosed inv(16) AML, Haferlach *et al. *[73] detected CBL splicing mutations in 6 patients (16%). The prevalence and features of these class I mutations are summarized in Table 2.

## Class II mutations (RUNX1 mutations, C/EBPα mutations and MLL rearrangement)

### RUNX1 mutations

Runx1 is required for definitive hematopoiesis and is necessary for the differentiation of myeloid progenitor cells to granulocytes [74]. Recently, Gaidzik *et al. *[75] precisely studied the frequency, biologic features and clinical relevance of Runx1 mutations in AML. They found that *RUNX1 *gene mutations, which span exons 3, 4, 5 and 8, were present in 53 of 945 patients (5.6%) with AML. RUNX1 mutations were associated with MLL-PTD (p = 0.0007) and IDH1/IDH2 mutations (p = 0.03), inversely correlated with NPM1 (p < 0.0001), and in trend with CEBPα (p = 0.10) mutations. RUNX1 mutations predicted resistance to chemotherapy, as well as inferior EFS (p < 0.0001), RFS (p = 0.022) and OS (p = 0.051). In multivariate analyses, RUNX1 mutations were an independent prognostic marker for shorter EFS (p = 0.007) (Table 3). Tang *et al. *[76] examined 470 adult patients with *de novo *non-M3 AML. Among these patients, 63 distinct RUNX1 mutations were identified in 62 patients (13.2%). They also revealed that the mutations were positively associated with MLL-PTD but negatively associated with C/EBPα and NPM1 mutations. Schnittger *et al. *[77] detected 164 RUNX1 mutations in 147 of 449 patients (32.7%) with a normal karyotype or noncomplex chromosomal imbalances. RUNX1 mutations were most frequent in AML M0 (65.2%) followed by M2 (32.4%) and M1 (30.2%). The molecular genetic markers most frequently associated with RUNX1 mutations were MLL-PTD (19.7%), FLT3-ITD (16.3%) and NRAS mutations (9.5%). Multivariate analyses showed that RUNX1 mutations independently predicted an unfavorable prognosis for OS (p = 0.029).

**Table 3:** Clinical features of gene mutations in AML (class II mutations)

Gene	Clinical Features	**Selected Ref**.	Frequency
Runx1	In multivariable analysis, RUNX1 mutations were an independent prognostic marker for shorter EFS. Independent unfavorable prognosis for OS for RUNX1 mutation.	[75-77]	5.6% in AML 13.2% in de novo non-M3AML 32.7% in CN or noncomplex karyotype AML
C/EBPα	C/EBPα mutations had a trend for a better CR rate and significantly greater 5-year rates of EFS, DFS and OS.	[79,80]	5-14% in AML
MLL rearr.	Patients with MLL rearrangement had a lower EFS and a higher probability of relapse than MLL wild type patients.	[81]	4-14% in AML

### C/EBPα mutations

The transcription factor C/EBPα is crucial for the differentiation of granulocytes. C/EBPα inactivation may take place by acquired mutations, which may occur along the entire coding region. These mutations lead to increased translation of an alternative 30-kDa form with dominant negative activity on the full-length 42-kDa protein when they occur in the N-terminus, while C-terminal mutations result in deficient DNA binding and/or homodimerization activities [63,78]. As reviewed by Pabst and Mueller [79], mutations in the various portions of C/EBPα have been reported to occur in 5-14% of AML patients. Among patients with C/EBPα mutations, 91% were in the CN-AML molecular high-risk group (FLT3-ITD-positive and/or NPM1-wild-type), although they seemed to be associated with a good prognosis in AML with an intermediate-risk karyotype [80]. Compared with C/EBPα-wild-type patients, patients with C/EBPα mutations showed a trend for a better CR rate (93% vs. 77%; p = 0.06) and significantly higher 5-year rates of EFS (55% vs. 17%; p < 0.001), DFS (53% vs. 23%; p = 0.001) and OS (58% vs. 27%; p = 0.002) [80] (Table 3). However, the reason why C/EBPα mutations confer a good prognosis remains unclear.

### MLL rearrangement

Approximately 4-11% of patients with AML present with rearrangement of the MLL (also known as ALL1 or HRX) gene as the result of a PTD within a single MLL allele [81]. This aberration tends to be frequent in AML patients without chromosomal abnormalities [81]. MLL is a 430-kDa transcription factor with a complex structure that includes three AT-hook domains for DNA binding, a methyltransferase homology domain and a SET domain [81]. MLL is necessary for the maintenance for HOX gene expression [82]. The MLL SET domain can bind to the promoters of HOX genes [83]. Munoz *et al. *[84] examined 93 adult patients with *de novo *AML for the incidence and clinical features of MLL-rearranged AMLs. As a result, they detected MLL rearrangement in 13 patients (14%). In the FAB classification, there was a significantly higher percentage of M5 subtypes in the MLL-rearranged group. The MLL-rearranged patients had lower EFS (p = 0.001) and a higher probability of relapse (p = 0.07) than MLL-wild-type patients. The clinical features of these RUNX1 and C/EBPα mutations and MLL rearrangement are shown in Table 3.

## Conclusions

The development of novel technologies has led to the identification of several important genetic mutations in AML. Along with the development of whole genome sequencing, it is probable that the major genetic aberrations have been almost completely identified. The next stage is to clarify the consequence of these molecular alterations, especially for the newly identified molecules. As described in this review, mutations in several newly identified genes, such as TET2 and IDH1/2, lead to the aberrant hypermethylation signature in AML cells [24]. In addition, there were alterations in the DNA methylation patterns and/or gene expression profiles, such as *HOXB *genes, in samples with DNMT3a mutations compared with those without such mutations [19]. Collectively, these recent findings strongly suggest a link between recurrent genetic alterations and aberrant epigenetic regulation, resulting in abnormal DNA methylation statuses in myeloid malignancies.

**Figure 2:**
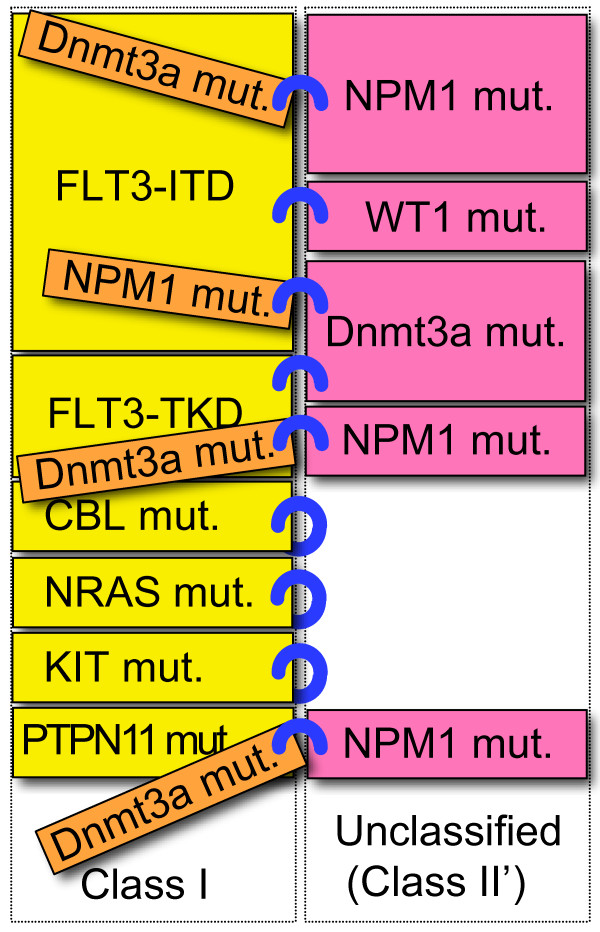
**The combination model of class I and unclassified mutations in AML**. Several unclassified mutations (NPM1, WT1, Dnmt3a) co-occur with several class I mutations. These may fall into putative class II mutations (termed "class II' mutations"), shown in pink boxes. Within these mutations, co-occurrences were observed between Dnmt3a, NPM1mutations (shown in orange boxes), therefore, these mutations may be exception of this model.

**Figure 3:**
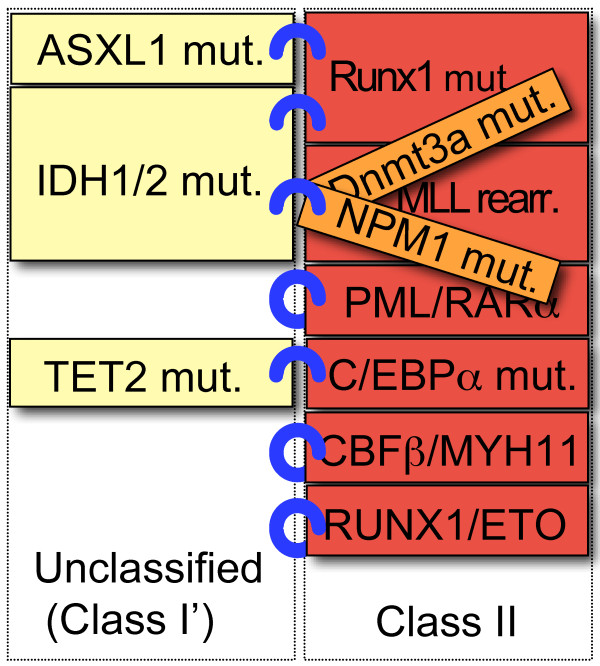
**The combination model of class II and unclassified mutations in AML**. Several unclassified mutations (ASXL1, IDH1/2, TET2) co-occur with several class II mutations. These may fall into putative class I mutations (termed "class I' mutations"), shown in light yellow boxes. IDH1/2 mutations co-occurred not only with MLL rearrangement, but also Dnmt3a and NPM1 mutations, as shown in orange boxes.

With the identification of novel genetic aberrations, increasing numbers of cooperative interactions of these genetic alterations have been discovered. These observations indicate that these unclassified mutations may fall into several subcategories. A revised model of these combinations, including "unclassified mutations" in AML is shown in Figure 2 and 3. In general, the mutations in the same category are mutually exclusive [14,85]. However, there are some exceptions. In "classical" class II mutations, co-occurrence was reported between RUNX1 mutations and MLL rearrangement [76] (Figure 1). Likewise, in unclassified mutations, co-occurrences were observed among Dnmt3a, NPM1 and IDH1/2 mutations [10,25,26]. Dnmt3a and NPM1 mutations co-occur with both classical class I and class II mutations, therefore, these mutations may not be simply categorized into "classical" class I or class II mutations, but it would fall into new category of mutations. Further characterization of these mutations, not only from clinical studies, but also from studies of transgenic animals may be needed.

For these several years, there are admirable progress for the identification of the molecular prognostic markers for CN-AML [86]. Based on these genetic alterations, several signal transduction pathway inhibitors and DNA methyltransferase inhibitors (decitabine, azacitidine) were reported for AML treatment [87]. The newly identified combinations of genetic aberrations will lead to a refined disease classification and to the development of more rational, epigenetic or signal transduction pathway-targeted therapies.

## Competing interests

The author declares that they have no competing interests.

## Authors' information

Professor and Chief, the Division of Molecular Hematology, Kitasato University Graduate School of Medical Sciences and the Division of Hematology, Kitasato University School of Allied Health Sciences, Sagamihara, Japan
